# APE1 stimulates EGFR-TKI resistance by activating Akt signaling through a redox-dependent mechanism in lung adenocarcinoma

**DOI:** 10.1038/s41419-018-1162-0

**Published:** 2018-10-31

**Authors:** Guo-Shou Lu, Mengxia Li, Cheng-Xiong Xu, Dong Wang

**Affiliations:** Cancer Center, Daping Hospital and Research Institute of Surgery, Third Military Medical University, Chongqing, 400042 China

## Abstract

Epidermal growth factor receptor tyrosine kinase inhibitors (EGFR-TKIs) have become the standard first-line treatment for advanced lung adenocarcinoma (LUAD) cancer patients with activating EGFR mutations. However, most patients show acquired resistance to EGFR-TKIs, thereby resulting in a modest overall survival benefit. Here, we found that expression level of APE1 was closely associated with TKI resistance in LUAD. Our clinical data show that level of APE1 was inversely correlated with progression-free survival rate and median time to progression in EGFR-mutated LUAD patients. Additionally, we observed increased expression of APE1 in TKI-resistant LUAD cell lines compared to their parental cell lines. Overexpression of APE1-protected TKI-sensitive LUAD cells from TKI-induced cell growth inhibition and cell death. In contrast, inhibition of APE1-enhanced TKI-induced apoptosis, cell growth inhibition and tumor growth inhibition in TKI-resistant LUAD. In addition, we identified that APE1 positively regulates Akt activation and APE1 overexpression-induced TKI resistance was attenuated by inhibition of Akt activity. Finally, we demonstrated that inhibition of the redox function of APE1 enhances the sensitivity of TKI-resistant LUAD cells to TKI treatment and inhibits Akt phosphorylation in TKI-resistant LUAD cells, but not by inhibition of the APE1 DNA repair function. Taken together, our data show that increased expression of APE1 significantly contributes to TKI resistance development in LUAD, and targeting APE1 may reverse acquired resistance of LUAD cells to TKI treatment. Additionally, our data show that APE1 regulates TKI resistance in LUAD cells by activating Akt signaling through a redox-dependent mechanism.

## Introduction

Lung cancer is the leading cause of cancer-related mortality worldwide, and lung adenocarcinoma (LUAD) is the most common histologic subtype of lung cancer^1,2^. In LUAD, several oncogenic driver mutations have been detected, including K-Ras, epidermal growth factor receptor (EGFR), and BRAF mutations^2–4^, and these activating genetic mutations are now targets for kinase-inhibitor therapy^2,5^. Among them, EGFR is found in 10–40% LUAD patients, occurring most frequently in never-smokers and in East Asian populations^6–8^. Notably, EGFR tyrosine kinase inhibitors (TKIs) have become the standard first-line treatment for advanced lung cancer patients with activating EGFR mutations^9^. However, acquisition of resistance to these EGFR-TKIs is almost inevitable at a median of 9–13 months, resulting in a modest overall survival benefit^10^.

T790M secondary mutation of EGFR is the most common acquired resistance mechanism to first-generation and second-generation EGFR-TKIs that account for approximately 50% of EGFR-TKI resistance cases of lung cancer^11^. Additional mechanisms of acquired resistance to EGFR-TKIs include activation of insulin-like growth factor-1 receptor (IGF-1R), amplification of MET and HER2, upregulation of the AXL receptor or its ligand, activating mutations in PIK3CA and BRAF, and SCLC transformation^6,10,11^. However, the TKI resistance mechanism for 15–30% of cases is still unknown^6,10,11^.

Apurinic/apyrimidinic endonuclease/redox factor-1 (APE1/Ref-1) is a multifunctional protein that plays critical roles both as a redox regulator of transcription factor activation and as part of the DNA damage response. Previous studies show that elevated APE1 significantly contributes to the development of therapeutic resistance and is positively correlated with poor clinical outcomes in several cancers^12^. Interestingly, although not in lung cancer, a recent report show that APE1 was involved in EGFR activation^13^. In addition, studies show that APE1 also involved in regulation of Akt activation^14,15^. Akt (protein kinase B) is a serine/threonine protein kinase that plays a key role in cancer by stimulating cell proliferation, inhibiting apoptosis, and modulating protein translation^16^. Notably, studies show that activated Akt signaling is involved in the therapeutic resistance of lung cancer, including both T790M and non-T790M mutation mechanisms of EGFR-TKIs resistance^5,17^. These findings suggest that APE1 may be involved in EGFR-TKIs resistance. However, the effects of APE1 on EGFR-TKIs resistance is unknown.

In this study, we identified that APE1 expression was increased in EGFR-TKI-resistant LUAD cell lines compared to their parental cell lines, and the level of APE1 was inversely correlated with median progression time in LUAD patients with EGFR mutations treated only with TKIs. Overexpression of APE1 reduced the sensitivity of to TKIs treatment in TKI-sensitive LUAD cells, while inhibition of APE1 enhanced sensitivity to TKI treatment in TKI-resistant LUAD cells. In addition, we identified that APE1-induced TKI resistance in LUAD cells by activating Akt signaling through a redox-dependent mechanism.

## Results

### Increased expression level of APE1 was associated with TKIs resistance in EGFR-mutated LUAD

**Fig. 1:**
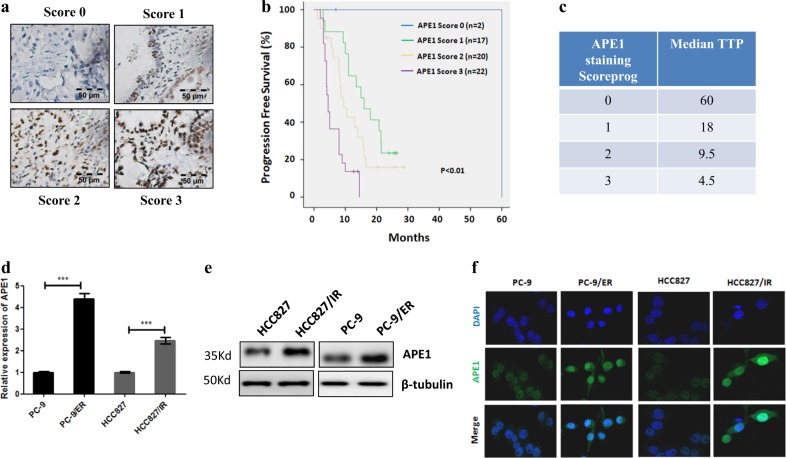
The level of APE1 was closely associated with TKI resistance in LUAD. **a** The expression of APE1 in LUAD specimens was measured by immunohistochemistry. **b** Kaplan–Meier analysis of progression-free survival rate for EGFR-mutated LUAD patients. Patients were divided into four groups based on APE1 staining score. **c** Median time to progress analysis. **d** qRT-PCR results show that the mRNA levels of APE1 were increased in TKI-resistant LUAD cell lines compared to their parental cell line. **e** Western blot analysis results show that protein levels of APE1 were increased in TKI-resistant LUAD cell lines compared to their parental cell line. **f** Immunofluorescence assay shows that protein levels of APE1 were increased in TKI-resistant LUAD cell lines compared to their parental cell line. HCC827/IR, Icotinib-resistant cell HCC827; PC-9/ER, Erlotinib-resistant cell PC-9; TTP time to progression; ***p < 0.001

To investigate the effect of APE1 expression levels on TKI treatment of LUAD patients with EGFR mutations, patients who were treated only with TKIs were divided into four groups based on APE1 staining score (Fig. 1a). In EGFR-mutated LUAD patients, APE1 levels were not associated with patient age, gender, smoking status, and TNM stage (Table 1). However, our data show that APE1 expression level were inversely correlated with progression-free survival rate (Fig. 1b) and median time to progression (TTP) in LUAD patients with EGFR mutations (Fig. 1c). In addition, we demonstrated significantly increased expression levels of APE1 in TKI-resistant LUAD cell lines HCC827/IR (resistant to icotinib) and PC-9/ER (resistant to erlotinib) (Supplement Fig. 1) compared to their parental cells at both mRNA (Fig. 1d) and protein levels (Fig. 1e). These results were further confirmed by immunofluorescence in TKI-resistant LUAD cells and their parental cells, and we observed similar results by Western blot analysis (Fig. 1f). Together, these data suggest that increased expression level of APE1 is closely correlated with TKI resistance in EGFR-mutated LUAD.

**Table 1:** Characteristics of lung adenocarcinoma patients with EGFR mutations

Variable	Number of patients				*p*
APE1 staining	Score 0	Score 1	Score 2	Score 3	
Gender					0.65
Male	1	7	12	13	
Female	1	10	8	9	
Age					0.94
60>	1	10	11	14	
≤60	1	7	9	8	
Smoking					0.45
Yes	0	4	8	9	
No	2	13	12	13	
T status					0.33
T1	0	2	0	1	
T2	1	9	6	6	
T3	1	2	5	3	
T4	0	4	9	12	
N status					0.38
N0	1	0	1	3	
N1	0	1	4	2	
N2	1	13	12	14	
N3	0	3	3	3	
M status					0.89
M0	0	2	3	2	
M1	2	15	17	20	
Treated TKIs					0.23
Gefitinib	1	8	5	3	
Erlotinib	1	9	13	15	
Icotinib	0	0	2	4	

### Overexpression of APE1 contributes to TKI resistance in EGFR-mutated LUAD

**Fig. 2:**
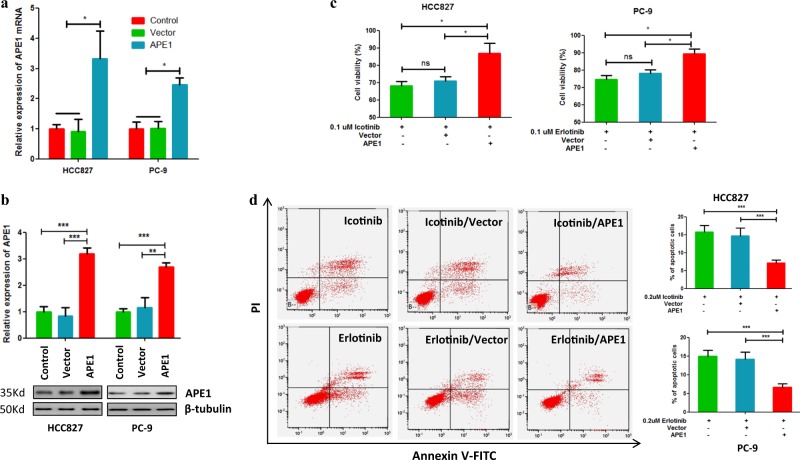
Overexpression of APE1-induced TKIs resistance in TKI-sensitive LUAD cells. **a**, **b** APE1 expression was significantly increased by transfection with an APE1-expressing plasmid at both mRNA and protein level in LUAD cells. Indicated cells were transfected by an APE1-expressing plasmid or control vector. After 48 h of transfection, cells were subjected to qRT-PCR and Western blotting. **c** Overexpression of APE1 significantly inhibited TKI-induced cell growth inhibition in TKI-sensitive LUAD cell lines. Indicated cells were transfected with indicated plasmids. After 24 h of transfection, cells were treated with indicated TKIs for 48 h, then subjected to MTT assay. **d** Overexpression of APE1 significantly suppressed TKI-induced apoptosis in TKI-sensitive LUAD cell lines. Indicated cells were transfected with indicated plasmids. After 24 h of transfection, cells were treated with indicated TKIs for 24 h, then subjected to apoptosis analysis. ns no significance; *p < 0.05, ***p < 0.001

Next, to investigate whether the increased expression of APE1 is directly involved in EGFR-TKI resistance, we overexpressed APE1 in TKI-sensitive LUAD cell lines HCC827 and PC-9 (Fig. 2a, b), and the cells were subsequently treated with TKIs and subjected to cell viability and apoptosis assays. Cell viability assay results show that APE1 overexpression significantly protected HCC827 and PC-9 cells from icotinib-induced and erlotinib-induced cell growth inhibition, respectively, compared to their control groups (Fig. 2c). Similarly, apoptosis assay results also show that overexpression of APE1 significantly protected HCC827 and PC-9 cells from icotinib-induced and erlotinib-induced cell death, respectively (Fig. 2d). Altogether, these data demonstrate that overexpression of APE1 significantly contributes to the development of TKI resistance in LUAD cells by attenuating TKI-induced apoptosis.

### Silencing of APE1 enhances the sensitivity of TKI-resistant LUAD cells to TKI treatment

**Fig. 3:**
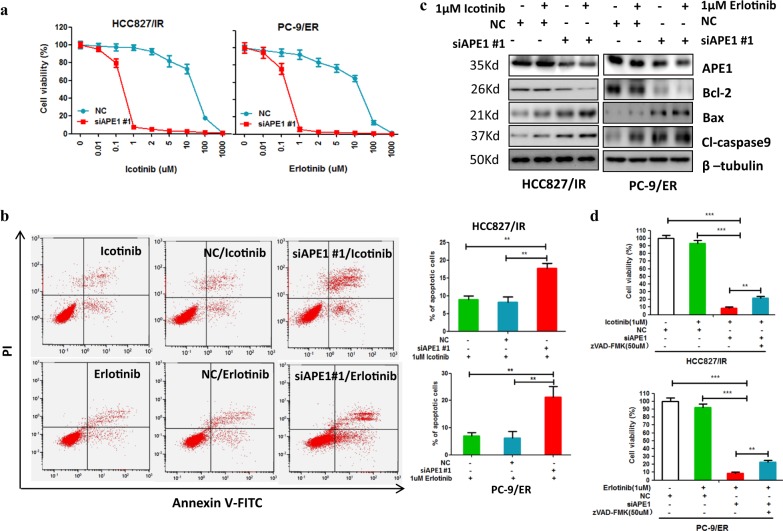
Silencing of APE1 enhanced the sensitivity of TKI-resistant LUAD cells to TKIs treatment. **a** Silencing of APE1 significantly enhanced TKI-induced cell growth inhibition in TKI-resistant LUAD cells. Indicated cells were transfected with APE1 siRNA or negative control nucleotides (NC). After 24 h of transfection, cells were treated with indicated TKIs for 72 h, and cells were then subjected to cell viability assay. **b** Silencing of APE1 significantly increased TKIs-induced apoptosis in TKI-resistant LUAD cells. HCC827/IR and PC-9/ER cells were transfected with or without APE1 siRNA. After 24 h of transfection, cells were treated with indicated TKIs for 48 h, and cells were then subjected to flow cytometry assay. **c** Combination treatment of APE1 silencing and TKI significantly increased pro-apoptotic protein expression while suppressing the expression of anti-apoptotic proteins in TKI-resistant LUAD cells. Indicated cells were transfected with siRNA of APE1 or negative control oligonucleotides (NC), then treated with indicated TKIs. After 48 h of TKI treatment, cells were subjected to Western blotting. **d** Caspase inhibitor zVAD-FMK treatment inhibited silencing of APE1 enhanced cell viability inhibition effects of TKIs in TKI-resistant LUDA cells. Indicated cells were transfected with siRNA of APE1 (siAPE1) or negative control oligonucleotides (NC). After 24 h of transfection, cells were reseed in 96-well plates. After overenight, cells were treated with indicated drugs for 48 h, then subjected to cell viability assay. **p < 0.01

**Fig. 4:**
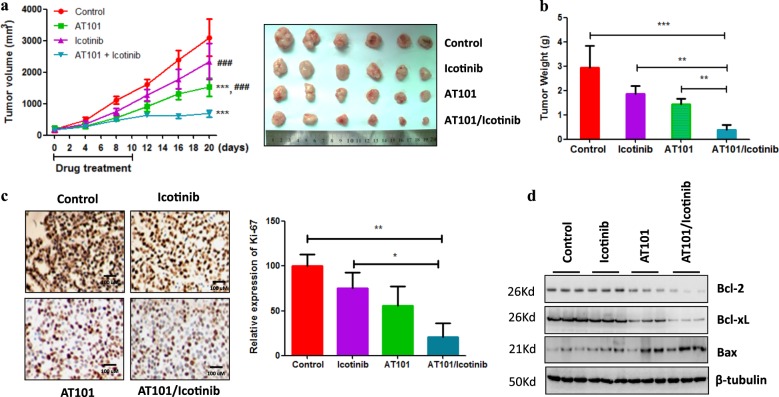
Combination treatment of Icotinib and AT101 dramatically inhibited TKI-resistant tumor growth in a xenograft model. **a** Tumor growth curve. Xenograft models are generated by HCC827/IR cells. When tumor volume reached approximately 100 mm^3^, mice were treated with Icotinib (4 mg/kg, IP) or AT101 (35 mg/kg, oral injection) or combination for 10 days. Mice were killed 10 days after drug treatment. Asterisk indicates compared to control group; hash indicates compared to combination treatment group. ***p < 0.001; ^###^*p* < 0.001. **b** Tumor weight. At the end of the animal experiment, tumors were collected from mice and weighed. **c** Combination of AT101 and Icotinib significantly inhibited cancer cell proliferation. Ki-67 expression was detected by immunohistochemistry. **d** Combination of AT101 and Icotinib significantly increased and inhibited pro- and anti-apoptotic protein expression, respectively

Our observation that overexpression of APE1 contributes to the development of TKI resistance in LUAD cells prompted us to investigate whether the silencing of APE1 could enhance the sensitivity of TKI-resistant LUAD cells to TKI treatment. As expected, our results show that silencing of APE1 dramatically enhanced icotinib-induced and erlotinib-induced cell growth inhibition in TKI-resistant LUAD cell lines HCC827/IR and PC-9/ER, respectively (Fig. 3a and Supplementary Fig. 2a). Additionally, APE1 silencing significantly stimulated icotinib-induced and erlotinib-induced apoptosis of TKI-resistant LUAD cell lines HCC827/IR and PC-9/ER, respectively (Fig. 3b and Supplementary Fig. 2b). Consistently, we detected significantly increased pro-apoptotic protein expression, including cleaved caspase-9 and Bax, while decreased anti-apoptotic Bcl-2 expression in the combination group of APE1 silencing and TKI treatment compared to the TKI only treatment group (Fig. 3c and Supplementary Fig. 2c), suggesting that silencing of APE1 sensitize TKI-resistant LUAD cells to TKI treatment partially through inducing apoptosis. In fact, MTT and apoptosis analysis showed that treatment of caspase inhibitor zVAD-FMK (carbobenzoxy-valyl-alanyl-aspartyl-[O-methyl]-fluoromethylketone) significantly inhibit silencing of APE1 enhanced cell viability inhibition (Fig. 3d) and apoptosis of TKIs (Supplementary Fig. 3a, b) in both HCC827/IR and PC-9/ER cell lines. Furthermore, we confirmed the in vitro results using a xenograft model generated by HCC827/IR cells. Here, we used AT101 as an APE1 inhibitor. Because AT101 is R-(-)-enantiomer of gossypol and our previous study show that gossypol can inhibits APE1 activity by directly interacting to APE1^18^. Furthermore, AT101 inhibited APE1 expression in gastric cancer^19^ and TKI resistance LUAD cell lines (Supplementary Fig. 4a). Notably, consistent with APE1 silencing, AT101 treatment can overcome APE1 overexpression-induced TKI resistance in LUAD cell HCC827 and PC-9 (Supplementary Fig. 4b). These findings clearly suggesting that AT101 is a inhibitor of APE1. Consistent with the in vitro results, animal experiments show that combination treatment of icotinib and APE1 inhibitor, AT101, significantly suppressed tumor growth (Fig. 4a, b) and cancer cell proliferation (Fig. 4c) compared to the control or single drug treatment group. Additionally, the combined treatment of icotinib and AT101 significantly induced pro-apoptotic protein Bax expression and inhibited anti-apoptotic protein Bcl-2 and Bcl-xL expression compared to control or single drug treatment group (Fig. 4d). Taken together, these findings suggest that inhibition of APE1 can overcome the resistance of TKI-resistant LUAD cells to TKI treatment.

### Akt pathway was involved in APE1-mediated EGFR-TKI resistance in LUAD cells

**Fig. 5:**
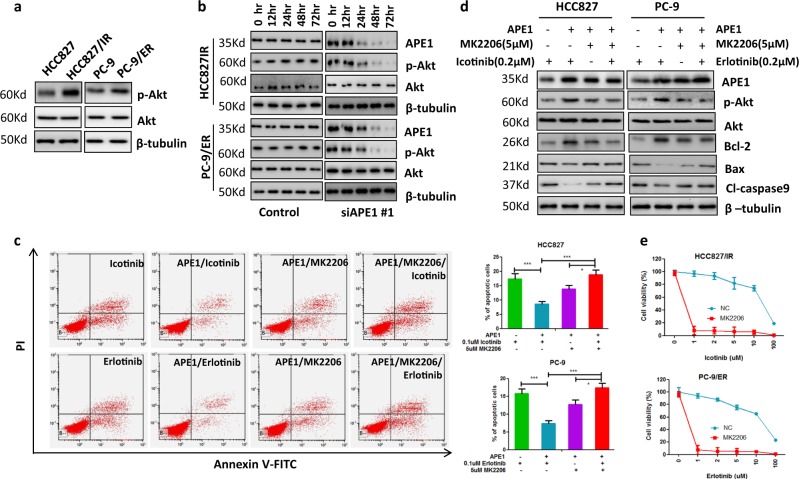
APE1 contributes to TKI resistance through activating Akt. **a** p-Akt was increased in TKI-resistant LUAD cells compared to their parental cells. **b** Silencing of APE1 significantly inhibited Akt phosphorylation in TKI-resistant LUAD cells. Indicated cells were transfected with APE1 siRNA, then subjected to Western blot analysis at indicated times. **c** Inactivation of Akt inhibited APE1 overexpression-caused inhibition of TKI-induced apoptosis in LUAD cells. Indicated cells were transfected with or without the APE1 expression plasmid. After 24 h of transfection, cells were treated with indicated drugs for 48 h, then subjected to apoptosis analysis. **d** Inactivation of Akt suppressed APE1 overexpression-induced stimulation of anti-apoptotic proteins’ expression and inhibition of pro-apoptotic proteins’ expression. Indicated cells were transfected with or without the APE1-expressing plasmid. After 24 h of transfection, cells were treated with indicated drugs for 24 h. Then, cells were subjected to Western blotting. **e** Treatment with Akt inhibitor significantly enhanced TKI-induced cell growth inhibition in TKI-resistant LUAD cells. Indicated cells were treated with indicated TKI and 5 µM of MK2206 for 72 h, then subjected to cell viability assay. *p < 0.05’; **p < 0.01; ***p < 0.001

Activated Akt signaling is involved in the development of EGFR-TKI resistance. Interestingly, previous studies show that APE1 is involved in the activation of Akt signaling^15,20^. Thus, we subsequently assessed the Akt and p-Akt expression levels in both TKI-resistant and their parental cells. As shown in Fig. 5a, total Akt levels were not upregulated in TKI-resistant cells, while p-Akt levels were significantly upregulated in both TKI-resistant cell lines HCC827/IR and PC-9/ER compared to their corresponding parental cells. Furthermore, to investigate whether increased levels of p-Akt were associated with APE1, we silenced APE1 in TKI-resistant cells and then measured total Akt and p-Akt expression by Western blotting. The results show that silencing of APE1 dramatically decreased the level of p-Akt in both HCC827/IR and PC-9/ER cells compared to their corresponding control cells, but total Akt levels did not change with APE1 silencing (Fig. 5b). Next, to investigate whether Akt activation is involved in the development of APE1-induced TKI resistance, APE1 was overexpressed in HCC827 and PC-9 cells, and the cells were then treated with TKI with or without Akt inhibitor MK2206. The results show that inhibition of Akt significantly attenuated APE1 overexpression-induced inhibition of TKI-caused apoptosis in both HCC827 and PC-9 LUAD cell lines (Fig. 5c). In addition, Western blot analysis showed that MK2206 treatment inhibited APE1 overexpression-induced Akt phosphorylation, anti-apoptotic protein Bcl-2 expression while increasing APE1 overexpression-suppressed expression of pro-apoptotic proteins, Bax and cleaved caspase-9 (Fig. 5d). Consistent with these data, the cell viability assay showed that MK2206 treatment significantly enhanced the sensitivity to TKI treatment of TKI-resistant LUAD cells (Fig. 5e). Taken together, these results suggest that APE1 induces TKI resistance partially through activation of Akt in LUAD.

### APE1 induces TKI resistance through a redox-dependent mechanism in LUAD

**Fig. 6:**
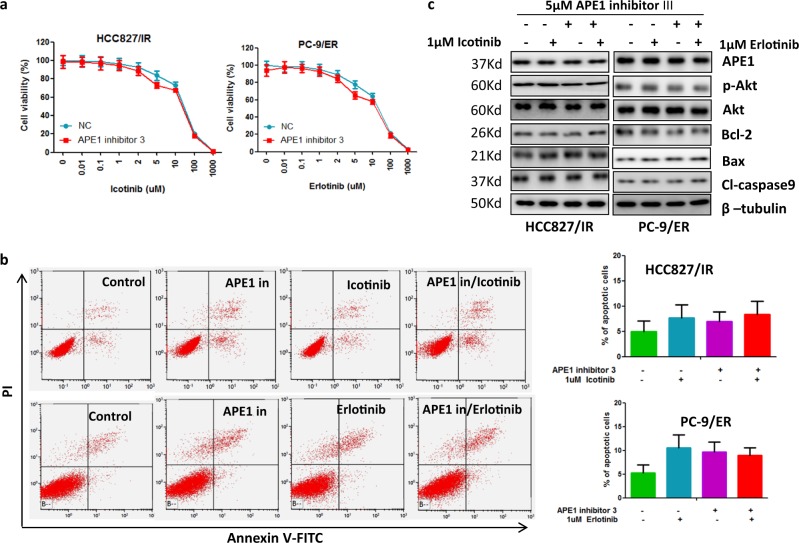
Inhibition of APE1 DNA repair function did not affect Akt phosphorylation and sensitivity of TKI-resistant LUAD cells to TKI treatment. **a** APE1 inhibitor III did not affect TKI-induced cell growth inhibition in TKI-resistant LUAD cells. Indicated cells were treated with indicated concentrations of TKI and 5 µM of APE1 inhibitor III for 72 h, then subjected to cell viability assay. **b** Combination treatment of APE1 inhibitor III and TKI did not significantly induce apoptosis in TKI-resistant LUAD cells. Indicated cells were treated with indicated concentrations of TKI and 5 µM of APE1 inhibitor III for 48 h, then subjected to apoptosis assay. **c** Combination treatment of APE1 inhibitor III and TKI did not affect apoptotic-related protein expression in TKI-resistant LUAD cells. Indicated cells were treated with 1 µM of indicated TKI and 5 µM of APE1 inhibitor III for 48 h, then subjected to Western blot analysis

**Fig. 7:**
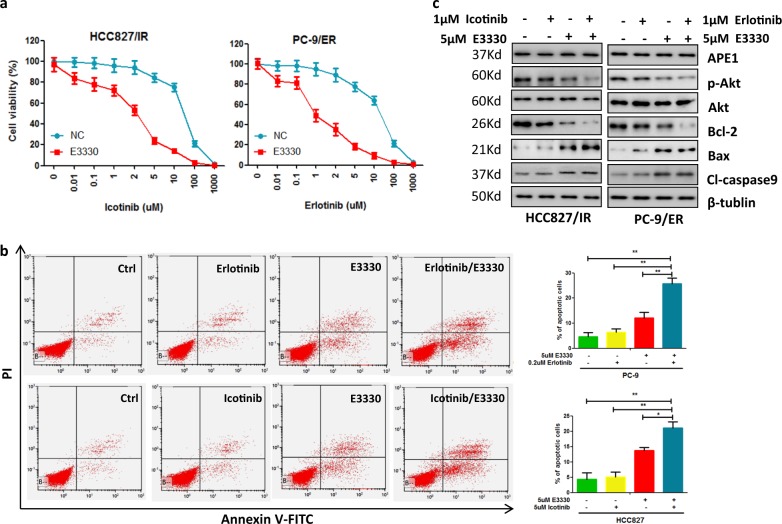
Inhibition of APE1 redox function inhibited Akt phosphorylation and enhanced the sensitivity of TKI-resistant LUAD cells to TKI treatment. **a** Treatment of APE1 redox function inhibitor E3330 significantly enhanced TKI-induced cell growth inhibition in TKI-resistant LUAD cells. Indicated cells were treated with indicated concentrations of TKI and 5 µM of E3330 for 72 h, then subjected to cell viability assay. **b** E3330 significantly enhanced TKI-induced apoptosis in TKI-resistant LUAD cells. Indicated cells were treated with indicated concentrations of TKI and 5 µM of E3330 for 48 h, then subjected to apoptosis assay. **c** Treatment of E3330 with or without TKIs inhibited Akt phosphorylation, anti-apoptotic proteins expression and stimulated pro-apoptotic proteins expression in TKI-resistant LUAD cells. Indicated cells were treated with 1 µM of indicated TKI and 5 µM of E3330 for 48 h, then subjected to Western blot analysis. **p < 0.01; ***p < 0.001

As described above, APE1 is a multifunctional protein that plays a role as a redox regulator of transcription factor activation and as an apurinic/apyrimidinic-site endonuclease. To investigate which function of APE1 was involved in Akt activation and development of TKI resistance, we treated TKI-resistant LUAD cells with APE1 redox function inhibitor, E3330, or APE1 DNA repair activity inhibitor, APE1 inhibitor III, and then checked the p-Akt levels and the sensitivity to TKI in TKI-resistant LUAD cells. Our results show that APE1 inhibitor III treatment did not affect TKI-induced cell growth inhibition (Fig. 6a), apoptosis (Fig. 6b) and apoptosis-related protein expressions (Fig. 6c). In contrast, redox inhibitor E3330 treatment significantly enhanced TKI-induced cell growth inhibition (Fig. 7a), apoptosis (Fig. 7b) and expression of pro-apoptotic proteins cleaved caspase-9 and Bax, while it inhibited expression of anti-apoptotic protein Bcl-2 (Fig. 7c) in TKI-resistant LUAD cells. Consistent with these results, APE1 inhibitor III treatment did not change p-Akt levels (Fig. 6c), but E3330 treatment significantly inhibited p-Akt levels in TKI-resistant LUAD cells (Fig. 7c). These findings suggest that APE1 induces TKI resistance and Akt activation through an APE1 redox-dependent mechanism in LUAD.

## Discussion

EGFR-TKIs are widely used for the treatment of EGFR-mutated LUAD patients. Thus, the occurrence of TKI resistance is a crucial clinical issue with respect to the treatment of LUAD with EGFR mutations^21^. In the present study, we used clinical sample analyses and a series of in vitro and in vivo experiments to identify that increased expression of APE1 was closely associated with TKI resistance development in LUAD. Our results show that APE1 was significantly increased in TKI-resistant LUAD cells compared to their parental cells, and EGFR-mutated LUAD patients with high expression APE1 presented lower progression-free survival rates and shorter median time to progression compared to patients with low APE1 expression when treated with TKIs. Overexpression of APE1 inhibits TKI-induced cell growth inhibition and cell death in TKI-sensitive LUAD cells, while silencing of APE1 significantly stimulated TKI-induced cell growth inhibition and apoptosis in TKIs-resistant LUAD cells. Consistent with in vitro, in a xenograft model generated by TKI-resistant LUAD cells, combination treatment of APE1 inhibitor and TKI markedly inhibited tumor growth compared to the control and single drug treatment groups. Taken together, these findings suggest that increased expression level of APE1 significantly contributes to TKI resistance development, and APE1 may be an ideal target for part cases with acquired EGFR-TKIs resistance.

In this study, we also clarified the mechanisms of APE1 on the regulation of sensitivity to TKIs in LUAD cells. The results of the present study indicated that APE1 contributes to TKI resistance development by activating Akt signaling in LUAD cells. Our data show that p-Akt was increased in TKI-resistant LUAD cells and overexpression of APE1 increases p-Akt level in LUAD cells. In addition, increased expression of p-Akt in TKI-resistant LUAD cells was significantly reduced by silencing APE1. Furthermore, the inhibition of Akt activation significantly inhibited APE1 overexpression-induced TKI resistance in LUAD cells. These findings also suggest that inhibition of Akt activation may be an ideal target for APE1 overexpression in TKI-resistant LUAD.

Next, we demonstrated that APE1 induces Akt activation through a redox-dependent mechanism in LUAD cells. Previous studies show that APE1 is involved in the regulation of cell proliferation and apoptotic protein expression by redox-specific functions^22^. Consistent with this report, our results also show that APE1 has a redox function dependently involved in TKI resistance development and Akt activation. Our results show that inhibition of the APE1 redox function can inhibit APE1 overexpression-induced resistance of TKI and Akt activation in LUAD. However, inhibition of the APE1 DNA repair function does not affect APE1-induced TKI resistance and Akt activation in LUAD cells. However, the detailed mechanism of how APE1 regulates Akt activation needs further study.

In conclusion, we demonstrated that aberrant increased expression of APE1 contributes to development of EGFR-TKI resistance in LUAD with EGFR mutations by activating Akt signaling through a redox-dependent mechanism. Our data also provide a rationale for targeting APE1 in EGFR-resistant LUAD patients.

## Materials and methods

### Human specimens, cell culture, and transfection

Human specimens were collected from 61 LUAD patients with EGFR mutation by biopsy or surgery at Daping Hospital and Research Institute of Surgery, Third Military University. The patients‘ characteristics are summarized in Table 1. The collection and use of human samples were approved by the ethical review committees.

PC-9 and PC-9/ER (Erlotinib-resistant PC-9) were obtained from cancer center of Xinqiao Hospital, Third Military Medical University. HCC827 and HCC827/IR (Icotinib-resistant HCC827) cells were obtained from Cancer Institute, Peking Union Medical College, Chinese Academy of Medical Sciences. Cells were cultured in RPMI with 10% fetal bovine serum, 100 μg/me streptomycin and 100U/me penicillin at 37 °C. All materials for cell culture were purchased from Sigma-Aldrich (St. Louis, MO, USA).

Cell transfection was performed using Lipofectamine 3000 according to manufacture’s instruction (Invitrogen). APE1 expression vector was purchased from Genechem Co. (Shanghai, China) and siRNAs of APE1 were obtained from RiboBio Co. (Guangzhou, China). The APE1 siRNA target sequences are as follows. First siRNA of APE1 (siRNA of APE1 #1): GTTGGTTGGCGCCTTGATT; second siRNA of APE1 (siRNA of APE1 #2):GTTGGCGCCTTGATTACTT.

### Cell viability and flow cytometric analysis

Indicated cells were plated in a 96-well plate at a density of 5000 cells/well. After 12 h of cell seeding, cells were treated with indicated drugs for 72 h, then cell viability was determined using a CCK-8 Kit (Dojindo Laboratories, Kumamoto, Japan), according to the manufacture’s instruction.

Apoptotic cells were detected using flow cytometric analysis with the Annexin V-FITC kit (CalbioChem, Shanghai, China) according to the manufacturer’s instruction.

### RNA extraction and qRT-PCR

Total RNA was extracted using TRIzol reagent (Invitrogen) according to manufacture’s instruction. Reverse transcription (RT) and PCR were performed with a High-Capacity cDNA Reverse Transcription kit and a QuantiTect SYBR Green PCR kit (Qiagen), respectively. The primer sequences as below: APE1 forward, 5′-CCGAATTCATGCCGAAGCGTGGGA-3′; and reverse, 5′-CCGCTCGAGTCGCAGTGCTAGGTATAG-3′. GAPDH forward, 5′-GCAGGGGGGAGCCAAAAGGGT-3′; and reverse, 5′-TGGGTGGCAGTGATGGCATGG-3′.

### Western blot, immunohistochemistry, and immunofluorescence assay

Western blotting, immunofluorescence (IF)^23^, and immunohistochemistry (IHC)^24^ were performed as previously described. Antibodies against for APE1, p-AKT, total Akt, Bcl-2, Bcl-xL, cleaved caspase-9, Bax, β-tubulin, and Ki-67 were purchased from Abcam (Cambridge, MA, USA).

### Animal experiments

Animal experiments were conducted using 6-week-old female nude mice. 1 × 10^7^ HCC827/IR cells were in 100 μl of phosphate-buffered saline were subcutaneously injected into each mouse. When the mean tumor size reached approximately 100 mm^3^, mice were divided into four groups (6 mice per group). Control group mice were treated with PBS; Icotinib group mice were treated with Icotinib by IP injection (4 mg/day/Kg body weight) every day; AT101 group mice were treated with AT101 through oral injection every day (35 mg/Kg body weight/day); combination group mice were treated with Icotinib and AT101 every day as described above. The treatment of mice were conducted for 10 days and mice were sacrificed after 10 days of the end drug treatment. Tumor volumes were measured every 4 days.

## Electronic supplementary material

Supplementary Figures

Supplementary Figure legends
