# Supplementary material for: APE1 stimulates EGFR-TKI resistance by activating Akt signaling through a redox-dependent mechanism in lung adenocarcinoma
Source: Cell Death Dis. 2018 Oct 31;9(11):1111. doi: 10.1038/s41419-018-1162-0 (PMC6208429; doi:10.1038/s41419-018-1162-0)
Supplement: Supplementary file 1 — Supplementary Figures [file 41419_2018_1162_MOESM1_ESM.pptx]

## Slide 1
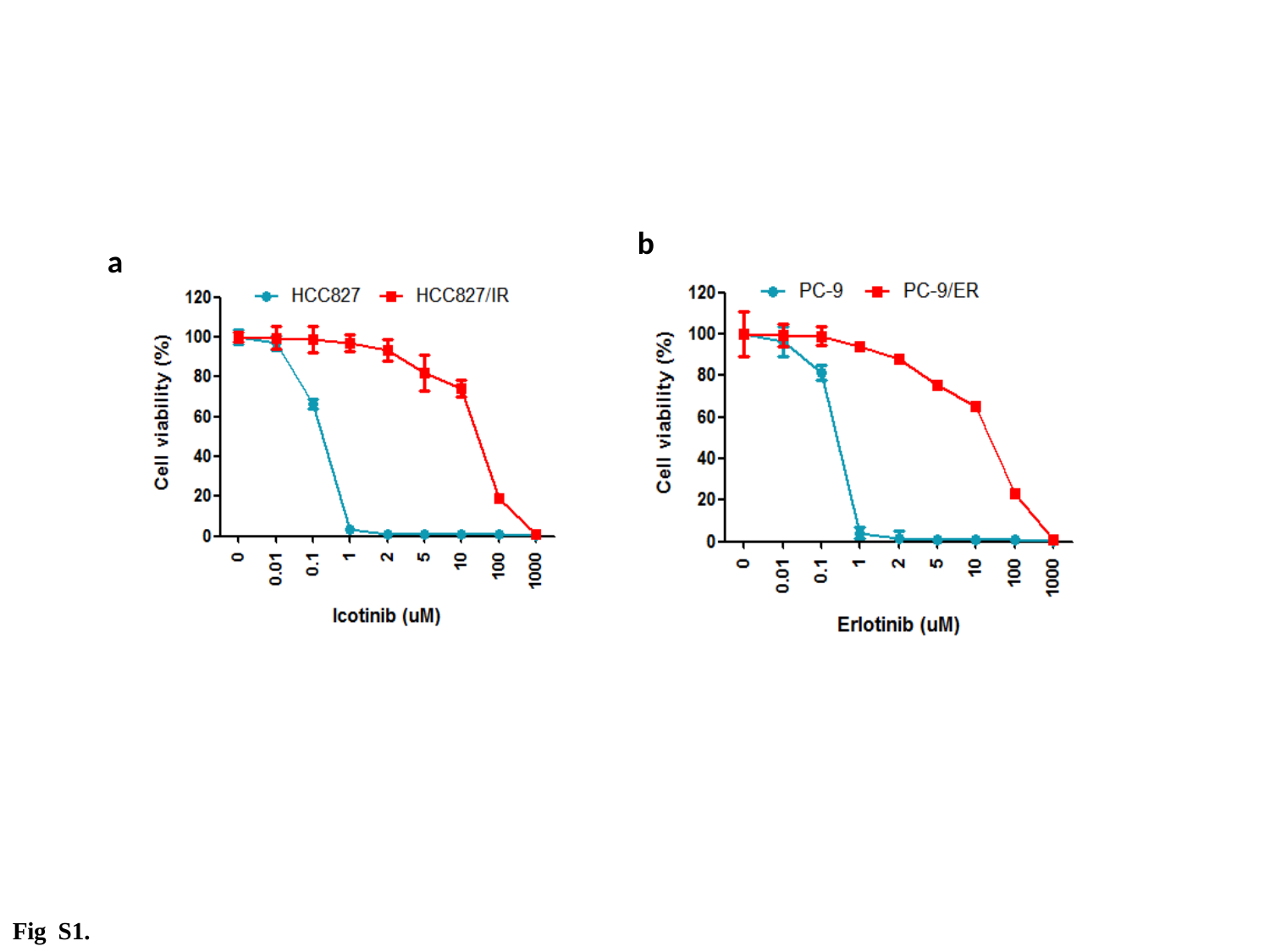

b
a
Fig S1.

## Slide 2
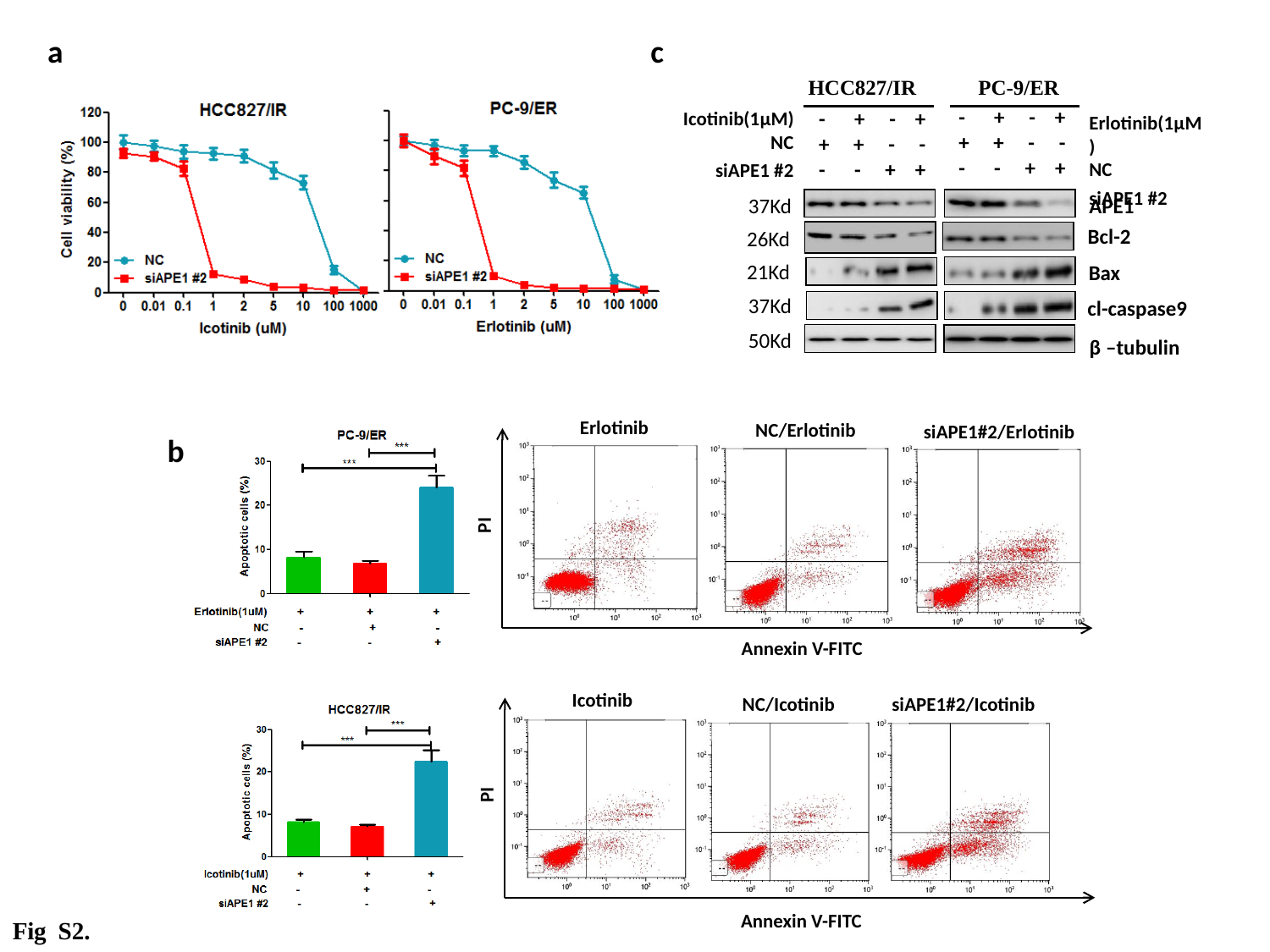

a
c
HCC827/IR
PC-9/ER
 - + - +
 + + - -
 - - + +
 - + - +
 + + - -
 - - + +
Icotinib(1μM)
NC
siAPE1 #2
Erlotinib(1μM)
NC
siAPE1 #2
37Kd
APE1
Bcl-2
26Kd
21Kd
Bax
37Kd
cl-caspase9
50Kd
β –tubulin
Erlotinib
NC/Erlotinib
siAPE1#2/Erlotinib
PI
Annexin V-FITC
b
Icotinib
siAPE1#2/Icotinib
NC/Icotinib
PI
Annexin V-FITC
Fig S2.

## Slide 3
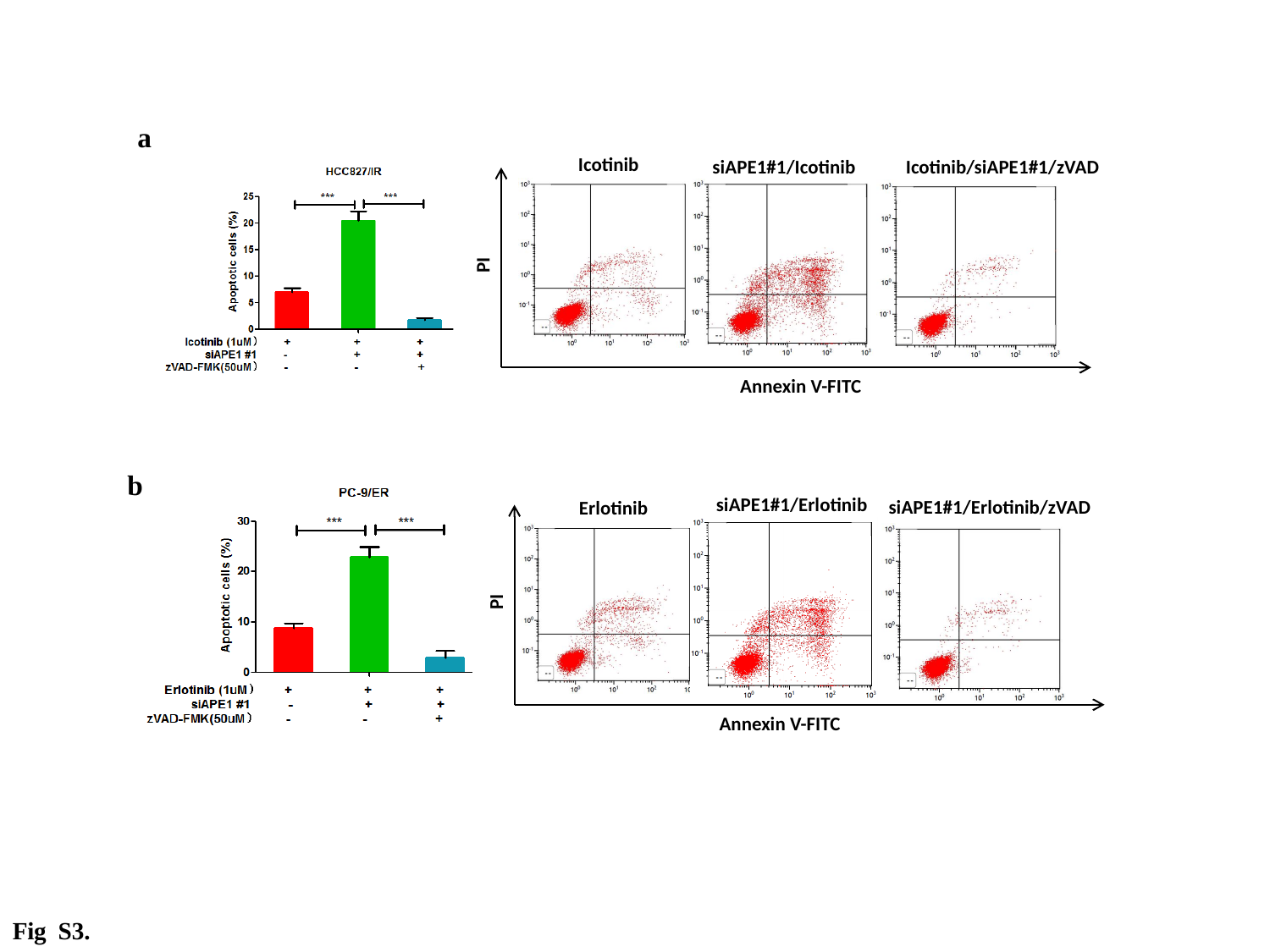

a
Icotinib
siAPE1#1/Icotinib
Icotinib/siAPE1#1/zVAD
PI
Annexin V-FITC
b
siAPE1#1/Erlotinib
siAPE1#1/Erlotinib/zVAD
Erlotinib
PI
Annexin V-FITC
Fig S3.

## Slide 4
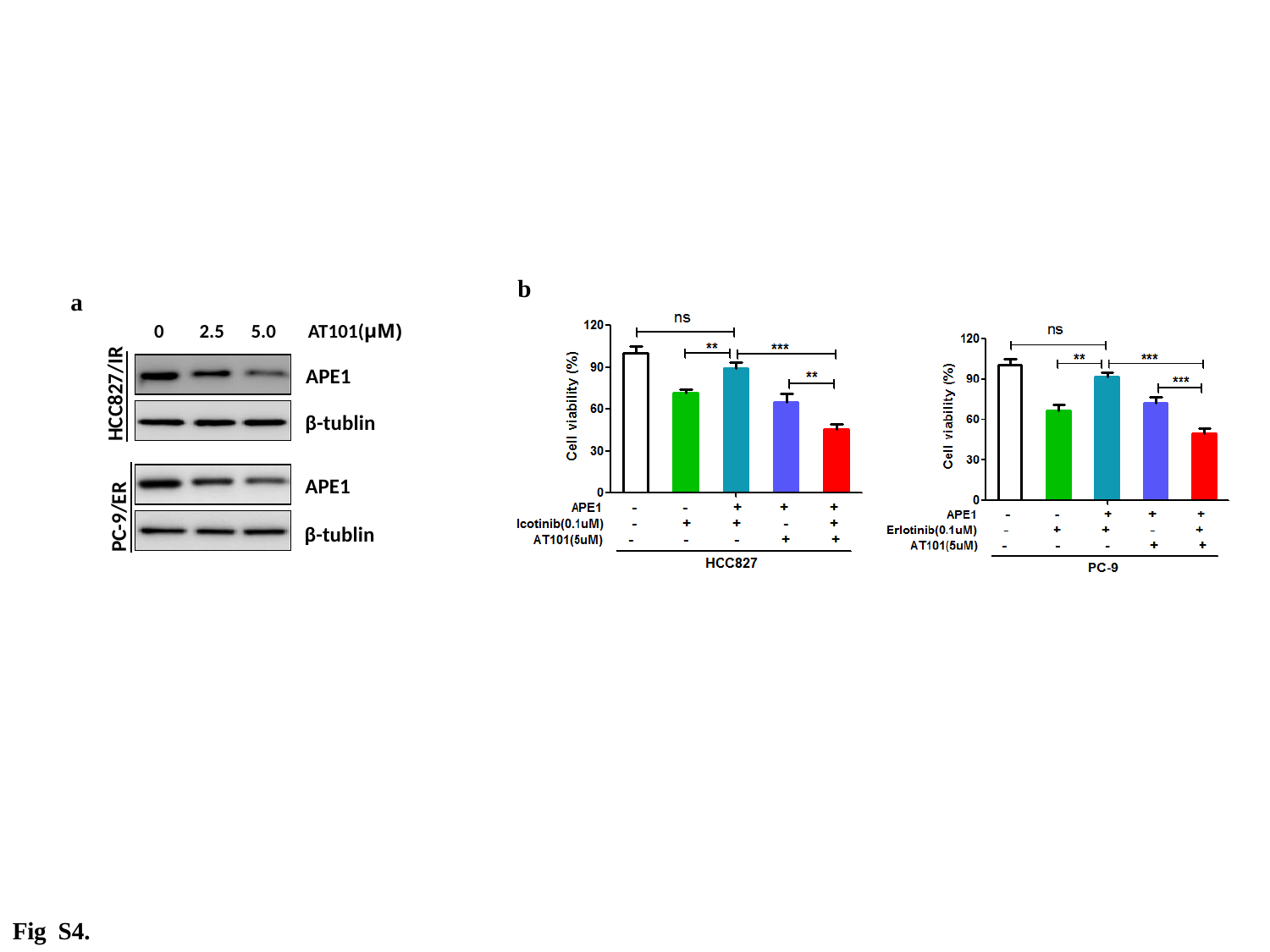

b
a
AT101(µM)
 0 2.5 5.0
APE1
HCC827/IR
β-tublin
APE1
PC-9/ER
β-tublin
Fig S4.
