# Supplementary material for: APE1 stimulates EGFR-TKI resistance by activating Akt signaling through a redox-dependent mechanism in lung adenocarcinoma
Source: Cell Death Dis. 2018 Oct 31;9(11):1111. doi: 10.1038/s41419-018-1162-0 (PMC6208429; doi:10.1038/s41419-018-1162-0)
Supplement: Supplementary file 2 — Supplementary Figure legends [file 41419_2018_1162_MOESM2_ESM.docx]

**Supplementary figure legend**

**Supplementary Fig. 1 The sensitivity of TKI-resistant NSCLC cell lines and their parental cell lines to TKIs treatment. a** HCC827/IR cells dramatically resistant to Icotinib treatment compared to their parental cell HCC827. Indicated cells were treated with indicated concentration of icotinib for 72 hours and then subjected to cell viability assay. **b** PC-9/ER cells presented resistant to erlotinib treatment. Indicated cells were treated with indicated concentration of erlotinib for 72 hours and then subjected to cell viability assay.

**Supplementary Fig. 2 Silencing of APE1 enhanced the sensitivity of TKI-resistant LUAD cells to TKIs treatment. a** Silencing of APE1 significantly enhanced TKI-induced cell growth inhibition in TKI-resistant LUAD cells. Indicated cells were transfected with APE1 siRNA or negative control nucleotides (NC). After 24 hrs of transfection, cells were treated with indicated TKIs for 72 hours, and then subjected to cell viability assay. **b** Silencing of APE1 significantly increased TKIs-induced apoptosis in TKI-resistant LUAD cells. HCC827/IR and PC-9/ER cells were transfected with or without APE1 siRNA. After 24 hrs of transfection, cells were treated with indicated TKIs for 48 hours, and then subjected to flow cytometry assay. **c** Combination treatment of APE1 silencing and TKI significantly increased pro-apoptotic protein expression while suppressing the expression of anti-apoptotic proteins in TKI-resistant LUAD cells. Indicated cells were transfected with siRNA of APE1 or negative control oligonucleotides (NC), then treated with indicated TKIs. After 48 hours of TKI treatment, cells were subjected to Western blotting. ***, p<0.001.

**Supplementary Fig. 3 Caspase inhibitor treatment significantly inhibited APE1 silencing enhanced apoptosis that induced by TKI in TKI-resistant LUAD cells. a** Caspase inhibitor zVAD-FMK treatment significantly inhibited APE1 silencing enhanced apoptosis that induced by Icotinib treatment in HCC827/IR cells. Cells were transfected with APE1 siRNA or negative control nucleotides (NC). After 24 hrs of transfection, cells were treated with Icotinib and/or zVAD for 72 hours, and cells were then subjected to cell viability assay. **b** Caspase inhibitor zVAD-FMK treatment significantly inhibited APE1 silencing enhanced apoptosis that induced by Erlotinib treatment in PC-9/ER cells. Cells were transfected with APE1 siRNA or negative control nucleotides (NC). After 24 hrs of transfection, cells were treated with Erlotinib and/or zVAD for 72 hours, and cells were then subjected to cell viability assay. ***, p<0.001.

**Supplementary Fig. 4 AT101 inhibits APE1 overexpression-induced TKI-resistance in TKI sensitive LUAD cell lines. a.** AT101 treatment inhibited APE1 expression in TKI-resistant cell line HCC827/IR and PC-9/ER. Indicated cells were treated with indicated concentration of AT101 for 48 hours, then subjected to Western blot analysis. **b.** AT101 inhibited APE1 overexpression-induced TKI-resistance in TKI sensitive LUAD cell lines. Indicated cells were transfected with APE1 expression plasmid or empty vector. After 24 hours of transfection, cells were re-plated in 96-well plate. 12 hours of re-plating, cells were treated with indicated drugs for 48 hours, then subjected to cell viability assay. ns, no significance; **, p<0.01; ***, p<0.001.
